# Monitoring Drought Stress in Common Bean Using Chlorophyll Fluorescence and Multispectral Imaging

**DOI:** 10.3390/plants12061386

**Published:** 2023-03-21

**Authors:** Tomislav Javornik, Klaudija Carović-Stanko, Jerko Gunjača, Monika Vidak, Boris Lazarević

**Affiliations:** 1 Centre of Excellence for Biodiversity and Molecular Plant Breeding (CoE CroP-BioDiv), Svetošimunska cesta 25, HR-10000 Zagreb, Croatia; 2 Department of Seed Science and Technology, Faculty of Agriculture, University of Zagreb, Svetošimunska cesta 25, HR-10000 Zagreb, Croatia; 3 Department of Plant Breeding, Genetics and Biometrics, Faculty of Agriculture, University of Zagreb, Svetošimunska cesta 25, HR-10000 Zagreb, Croatia; 4 Department of Plant Nutrition, Faculty of Agriculture, University of Zagreb, Svetošimunska cesta 25, HR-10000 Zagreb, Croatia

**Keywords:** high-throughput phenotyping, morphological changes, spectral reflectance, vegetation indices

## Abstract

Drought is a significant constraint in bean production. In this study, we used high-throughput phenotyping methods (chlorophyll fluorescence imaging, multispectral imaging, 3D multispectral scanning) to monitor the development of drought-induced morphological and physiological symptoms at an early stage of development of the common bean. This study aimed to select the plant phenotypic traits which were most sensitive to drought. Plants were grown in an irrigated control (C) and under three drought treatments: D70, D50, and D30 (irrigated with 70, 50, and 30 mL distilled water, respectively). Measurements were performed on five consecutive days, starting on the first day after the onset of treatments (1 DAT–5 DAT), with an additional measurement taken on the eighth day (8 DAT) after the onset of treatments. Earliest detected changes were found at 3 DAT when compared to the control. D30 caused a decrease in leaf area index (of 40%), total leaf area (28%), reflectance in specific green (13%), saturation (9%), and green leaf index (9%), and an increase in the anthocyanin index (23%) and reflectance in blue (7%). The selected phenotypic traits could be used to monitor drought stress and to screen for tolerant genotypes in breeding programs.

## 1. Introduction

Drought is found in all environments and it is considered as the most damaging environmental stress [1,2,3,4,5]. Even a brief water deficit leads to significant losses in crop yields [6,7] and hinders sustainable agricultural production worldwide [8,9,10]. Due to rapid climate changes, the frequency and duration of drought periods will increase [11,12]. Drought affects all essential physiological and developmental plant processes, such as photosynthesis, respiration, nutrient uptake, and the disproportionate relationship between roots and shoots [13]. It disrupts the normal function of the stomata, inhibits gas exchange and carbon assimilation, and leads to the overproduction of reactive oxygen species (ROS) and the development of oxidative stress [14]. The water deficit strongly inhibits cell division, the enlargement of the leaf surface, stem growth, and the multiplication of root cells [10]. All these factors lead to a decline in the productivity of photosynthesis (the basis for forming organic matter) and, ultimately, to a decline in yield [15].

Drought is one of the major disruptors of common bean (*Phaseolus vulgaris* L.) production, as about 60% of bean crops are grown in regions that suffer from water shortages [16]. The common bean is one of the most important grain legumes for human consumption [17]. In addition to its nutritional value, it also has a high economic value [18]. Developing drought-tolerant cultivars is needed to achieve sustainable common bean production in drought-prone environments and to provide economic benefits for producers and buyers. Traditional breeding programs are labor-intensive and require significant effort to isolate desirable from undesirable traits. The development of modern technologies such as high-throughput phenotyping (HTP), which combines different techniques to monitor the physiological response of plants to drought stress, accelerates and facilitates this type of research [19]. 

When starting a drought experiment, it is important to determine the timing to induce the water deficit and the stress intensity and duration [20]. There are many experimental setups to induce and monitor drought stress. The most commonly used are soil-based drought models [20,21], which are based on the gradual decline or immediate interruption of plant watering, and agar-based models (widely used in plant biology) [22,23]. Drought stress can be monitored by relative water content, osmotic adjustment, various physiological responses (efficiency of photosynthesis and stomatal conductance), and, increasingly, by chlorophyll fluorescence and multispectral imaging [24,25]. Because drought affects many plant traits on a whole plant scale, the utilization of HTP, which enables the simultaneous analysis of different plant traits, is of great value. Today, there are many HTP methods for drought stress detection, monitoring, and quantification [26]. The most widely used HTP methods are chlorophyll fluorescence analysis [27,28,29,30], multispectral analysis [31,32], 3D multispectral scanning [33,34], and thermal imaging [35,36]. These methods have many advantages, such as rapid data acquisition and processing, non-destructiveness, accurate insights into plant performance under different drought intensities and durations, excellent morphological and biochemical characterization of plants, etc. [26,37].

Many authors have successfully used different vegetation indices such as the normalized difference vegetation index (NDVI) [38] and chlorophyll fluorescence traits such as photochemical quenching (qP), non-photochemical quenching (NPQ) [39] and maximum quantum yield of PSII (Fv/Fm) [40] to assess drought stress in the common bean. Strong correlations were also found between growth (biomass) and vegetation indices (green normalized difference vegetation index (GNDVI)) when monitoring drought stress in the common bean [41]. In the case of the pinto bean, the GNDVI was also a good predictor of water stress [42]. Various multispectral analyses and vegetation indices have been successfully used to detect water stress in other crops. In the case of detecting drought stress in turfgrass, Hong et al. [43] concluded that weekly measurements with the NDVI, GNDVI, and blue normalized difference vegetation index (BNDVI) could improve irrigation management. However, experiments that combine several HTP techniques and enable simultaneous monitoring and comparison of different drought-induced morphological and physiological changes in the same experimental conditions are scarce. There is a necessity to use simple and reliable plant traits to screen large populations for drought tolerance.

In this study, we used HTP methods (chlorophyll fluorescence imaging, multispectral imaging, 3D multispectral scanning) to continuously monitor the occurrence of drought morphological and physiological symptoms during early and prolonged drought in the common bean. This experiment aimed to select the most drought-sensitive plant phenotypic traits which could be used for the early detection of drought stress and in screening for drought tolerance among common bean germplasms. 

## 2. Results

HTP techniques, including chlorophyll fluorescence imaging, multispectral imaging, and 3D multispectral scanning were used in this experiment to determine the occurrence of drought stress and its effects on the common bean over time. The results of the repeated measures of ANOVA and tests of differences between treatments within each measurement time are presented in Table S1. The least-square means and the pairwise differences comparison (Tukey’s HSD test) are shown in Table S2. 

### 2.1. Effect of Drought Stress on Morphological Traits

**Figure 1:**
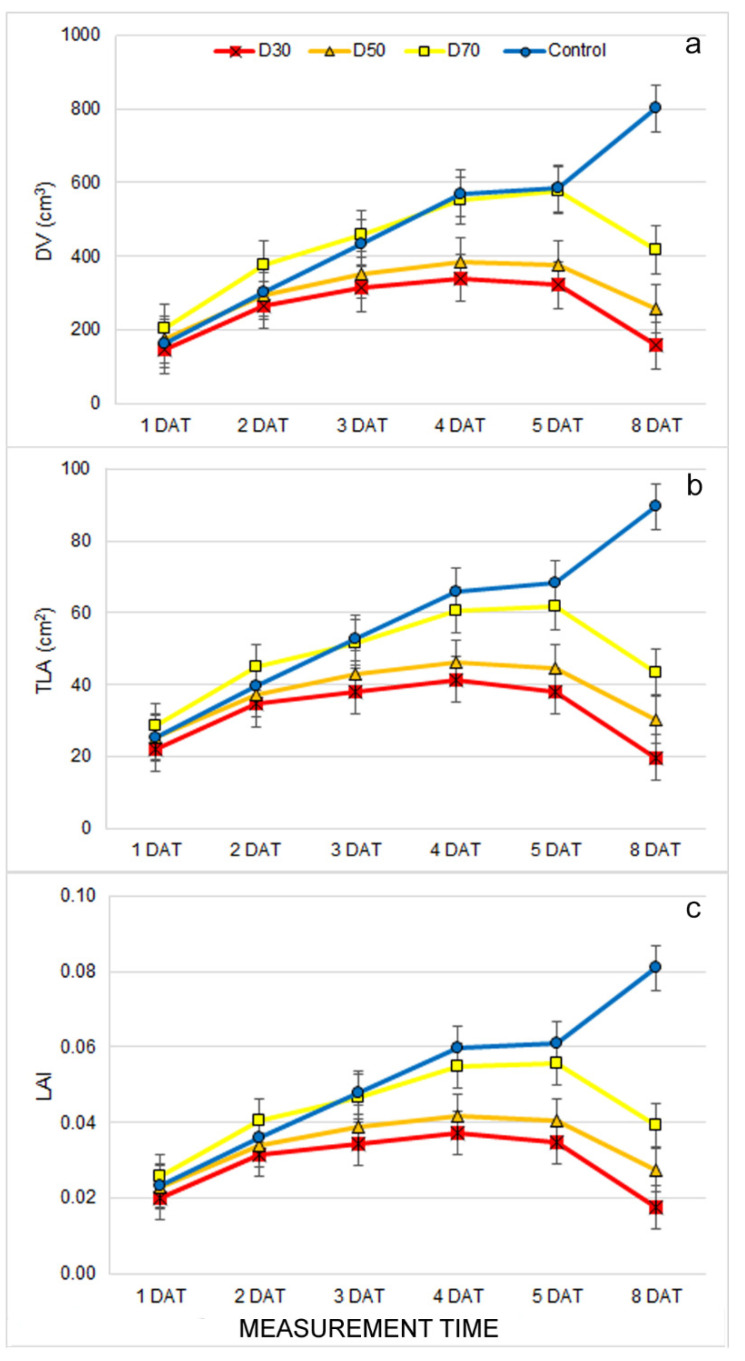
Mean ± double standard error (S.E.) for morphological traits: (**a**) digital volume (DV), (**b**) total leaf area (TLA), and (**c**) leaf area index (LAI) for plants grown under control and drought treatments (D30, D50, and D70) measured from the first to the eighth day after the beginning of treatments (1 DAT–8 DAT).

A significant effect of treatment × measurement time interaction (T × MT) was found for all measured morphological traits (Table S1). Examination of the difference among treatments within measurement time revealed that the earliest differences among treatments (already at 2 DAT) were found for leaf angle (LANG) and leaf inclination (LINC) (Table S1). However, these two traits were not consistently affected by drought treatments throughout the measurements; therefore, drought treatments had no distinct effect on these two traits. On the other hand, from 3 DAT, significantly lower digital volume (DV), total leaf area (TLA), and leaf area index (LAI) were found in D30 compared to D70 and the control treatments. From 7 DAT, these traits were also significantly lower for plants from D50 compared to D70 and the control (Table S2, Figure 1a–c). A significant difference in all morphological traits (except PH and LPD) between D70 and control plants was found at the last measurement (8 DAT) (Table S2, Figure 1a–c).

### 2.2. Effect of Drought Stress on Multispectral traits

**Figure 2:**
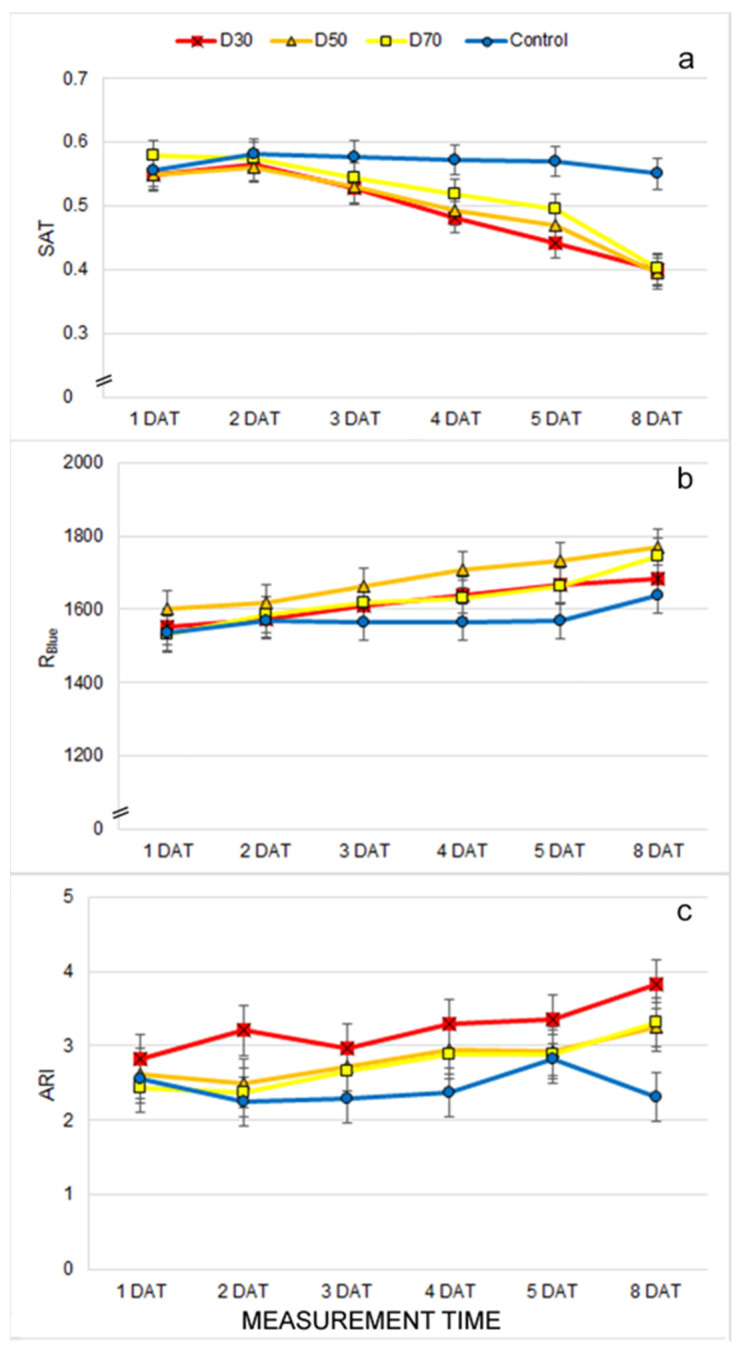
Mean ± double standard error (S.E.) for multispectral traits: (**a**) saturation (SAT), (**b**) reflectance in blue (R_Blue_), and (**c**) anthocyanin index (ARI) for plants grown under control and drought treatments (D30, D50, and D70) measured from the first to the eighth day after the beginning of treatments (1 DAT–8 DAT).

The evaluated multispectral parameters included reflectance in the visible (R_Red_, R_Green_, R_Blue,_ R_SpcGrn_, R_Chl_), the far-red (R_FarRed_), and near-infrared (R_NIR_) spectrum. In addition, these spectral reflections were used for the calculation of the vegetation indices (hue (HUE), saturation (SAT), value (VAL), chlorophyll index (CHI), anthocyanin index (ARI), green leaf index (GLI), normalized differential vegetation index (NDVI), and plant senescence reflectance index (PSRI)) (Table S4). For all measured and calculated multispectral traits, a significant effect of the T × MT interaction was found (Table S1). The earliest differences among drought treatments were found at 5 DAT for ARI and R_NIR_. At 2 DAT, a higher ARI was found in D30 compared to the control, and from 3 DAT, ARI was also higher for D50 compared to the control (Tables S1 and S2, Figure 2c). In contrast, R_NIR_ was not consistently affected by drought treatments during measurements (Tables S1 and S2). In addition, from 3 DAT, significantly higher GLI, SAT, and R_SpcGrn_ values were found in the control compared to D30, and significantly lower R_Blue_ was found in the control compared to D50 (Table S2, Figure 2a,b). After a prolonged drought, on the 8 DAT, the control significantly differed from the drought treatments in all multispectral traits except R_NIR_ and PSRI (Tables S1 and S2).

### 2.3. Effect of Drought Stress on Chlorophyll Fluorescence Traits

**Figure 3:**
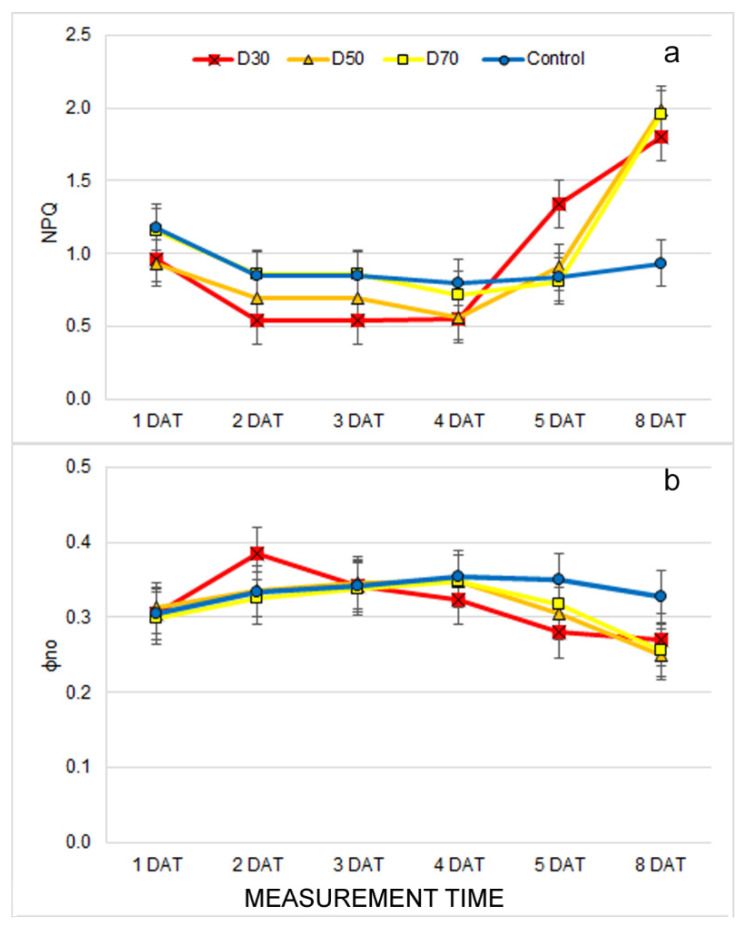
Mean ± double standard error (S.E.) for chlorophyll fluorescence traits: (**a**) non-photochemical quenching (NPQ), and (**b**) Quantum yield of non-regulated non-photochemical energy loss in PSII (ɸno) for plants grown under control and drought treatments (D30, D50, and D70) measured from the first to the eighth day after the beginning of treatments (1 DAT–8 DAT).

A significant interaction between drought treatments and measurement time (T × MT) was found for all measured chlorophyll fluorescence traits except the Fv/Fm, which was affected by both of the main effects of T and MT (Table S1). The earliest differences in chlorophyll fluorescence traits among drought treatments were found at 1 DAT (Fq′/Fm′and qP) (Tables S1 and S2). In addition, during subsequent measurements, there were significant differences among treatments for all measured chlorophyll fluorescence traits. However, these differences were inconsistent among drought treatments, which indicated that they were not solely affected by drought treatments. First, significant drought-induced differences, which showed consistency in the subsequent measurements, were found at 4 DAT when the NPQ increased, and ɸno decreases were found in D30 compared to other treatments (Tables S1 and S2, Figure 3a,b). A clear effect of drought treatments on traits showing photochemistry (Fq′/Fm′, qP and ETR) and an estimation of open PSII centers (qL) decreased significantly in all drought treatments compared to the control only after prolonged drought (8 DAT) (Table S2).

## 3. Discussion

When studying drought tolerance in plants, monitoring and controlling the level and onset of a water deficit is challenging. It is a complex process that takes into account both the plant’s water status and the available water in the soil [44]. Therefore, the design of the experiment and continuous measurements are of paramount importance to determine the occurrence of drought stress and the magnitude of its effect on the physiological changes that occur with the experimental plants [44,45]. For this experiment, we used washed river sand because of its low matrix potential, which allows for easy control of the water availability for plants. In addition, tall, narrow pots (in this case, tubes) and closely spaced plants were used to create a closed canopy that prevented excessive water evaporation from the sand surface [20]. Sand volumetric water content was continuously monitored throughout the experiment (Table S3). In addition, continuous HTP measurements were carried out throughout the experiment to detect the earliest drought-induced morphological and physiological symptoms. 

The earliest observed morphological changes caused by drought treatments were related to decreased leaf area (LAI and TLA) and DV. The reduced biomass and leaf area represent the adaptation of the plants to drought stress conditions [7,10]. Similar results were found in [46], where morphological traits of basil (digital volume and leaf area) were more reduced under drought than salinity stress. Moreover, a significant reduction in biomass after seven days of the drought was found in Arabidopsis (wild-type) and already after five days of drought (in mutant osca1) [30]. In this study with different irrigation treatments of the common bean, the LAI varied significantly. This suggests that water availability is one of the most important factors for crop development [47]. Because DV is calculated from total leaf area and plant height (which was not affected by drought treatments), it can be stated that the earliest and most pronounced effect of drought on the common bean morphology is reduced leaf area and LAI. Except for the TLA, LAI, and DV, we observed significant differences in the LANG and LINC among drought treatments. It is well known that drought causes plant wilting and, thus, changes in leaf angle [21]. However, these traits showed inconsistent results through different measurement times, which suggested that drought does not solely affect these traits. The possible cause of changing the LANG and LINC during different measurements could lie in the plant’s circadian rhythm, which changes the plant’s leaf position during the day [48]. Like morphological changes, drought treatments caused changes in leaf absorption/reflection properties. The earliest affected trait was the ARI which increased in treatment D30 compared to the control at 2 DAT. In addition, from 3 DAT, drought treatments caused a decrease in the GLI, SAT, and R_SpcGrn_ and an increase in R_Blue_ compared to the control. Although the increased reflection of visible light indicates a decrease in chlorophyll content and light absorption, our results showed no decrease in the CHI under drought treatments. Similarly, the NDVI, which is often considered as one of the best indices for monitoring plant water deficits [26,47], decreased only after prolonged drought in our study. Thus, the drought-induced increase in R_Blue_ is probably more related to an increased ARI (anthocyanin content) and less related to the decrease in chlorophyll content. Anthocyanins are dark blue pigments with a photoprotective role that increases biosynthesis, and concentrations are often reported under stressful conditions [6]. Other authors have reported similar results with different bean cultivars where reflectance values (visible spectrum, far red, and near infrared) were higher in treatments under full irrigation compared to treatments exposed to water stress [49]. An increase in anthocyanin content, reduced leaf area, and other structural and morphological changes probably play a role in protecting the light-harvesting apparatus and the photochemistry of the plants grown under drought treatments. The results of chlorophyll fluorescence imaging support this. Namely, chlorophyll fluorescence traits were found to be less affected by drought than morphological and multispectral traits, and significant changes caused by drought were only found after prolonged stress. Early differences in Fq′/Fm′ and qP, which were found at 1 DAT, and other differences were found in subsequent measurements, were inconsistent and were probably more affected by the above mentioned protective mechanisms in D30 and D50 and the continuation of young leaf growth and development in the control and D70 plants. It is well established that young developing leaves do not yet have developed photosystems, which are reflected as lower qL and, thus, lower photochemistry (qP, ETR, Fq′/Fm′) and higher non-photochemical energy dissipation (NPQ, qN) [50]. In our experiment, the earliest (4 DAT) affected chlorophyll fluorescence traits by drought treatments were increased NPQ and decreased ɸno, which indicated the activation of the photoprotective processes in the chloroplast [24]. Similar results of increased NPQ under drought were observed in lettuce seedlings [51], maize seedlings [52], common beans [39], rice, and wheat [53].

The result of this research shows that HTP methods can be used to detect and monitor early plant responses to drought. Our research enabled the extraction of the most responsive phenotypic traits under drought conditions. Namely, the earliest detected changes under drought stress were a decrease in the LAI and TLA, an increase in the ARI and R_Blue_, and a decrease in the GLI, SAT and R_SpcGrn_. In contrast, chlorophyll fluorescence traits were affected only after prolonged time and in severe drought conditions. Because drought can occur in different growth stages, can differ in duration and severity [8], and because this experiment was conducted under a controlled environment, these results should be confirmed under field conditions, at different growth stages, and using a higher number of genotypes to prove their agronomic relevance. 

## 4. Materials and Methods

### 4.1. Plant Material and Growth Conditions

Seeds of *Phaseolus vulgaris* cv. ‘Ferguson’, belonging to the ‘Cranberry’ type, were used for the experiment. Cultivar Ferguson is early harvest and bush growth (dwarf) bean variety. The seeds were sown in germination trays (containers) to obtain sufficient plant material for the experiment. Twelve days after germination, the uniformly developed seedlings were transplanted into 40 PVC tubes with a diameter of 5 cm, a height of 29 cm, and a volume of 569.4 cm^3^. Each PVC tube contained one plant and was filled with 785 g of washed and dried river sand (0.05–2.0 mm diameter size). During the germination phase and the experiment, the plants were kept in a growth chamber (2 m × 2.5 m = 5 m^2^) at 25/22 °C, a photoperiod of 16/8 h day/night, 60% relative air humidity, and a photosynthetic photon flux density (PPFD) of 250 µmol m^−2^ s^−1^, which was provided by Valoya L35, NS12 spectrum LED lights (Valoya Oy, Helsinki, Finland). 

### 4.2. Experimental Setup and Treatments

The experiment consisted of 4 treatments with 10 plants per treatment (40 plants in total) and was set up as a completely randomized block design (RBD) (the treatments were randomly allocated inside each block, and each block contained one plant per treatment). At the beginning of the experiment, all plants (treatments) were irrigated with 25 mL of a ½-strength Hoagland nutrient solution [54]. An additional 5, 25, or 45 mL of distilled water was added to the tubes giving D30 (30 mL), D50 (50 mL), and D70 (70 mL) drought treatments, respectively. The control tubes (with plants) were irrigated with 25 mL of a ½-strength Hoagland nutrient solution and with 25 mL of distilled water and were constantly supplied with water by immersing the base of the tubes (2 cm) in the distilled water during the experiment. This setup enabled evapotranspiration-driven capillary water to rise within the tubes. 

The experiment lasted eight days, and there was no additional irrigation of the plants in the drought treatments. The volumetric water content (VWC) of the sand was measured using a moisture meter with sand calibration, a HH2 Moisture Meter equipped with a WET sensor (type: WET-2) (Delta-T Devices Ltd., Cambridge, UK), and data are shown in Table S3.

### 4.3. HTP Measurements

A total of six measurements were performed, starting on the first day after the onset of treatments (1 DAT). Five consecutive measurements were performed during the next five days (1 DAT–5 DAT), and the last measurement was on the eighth day of the experiment (8 DAT). All measured plant traits, abbreviations, and devices used for the measurement are listed in Table S4. A detailed description of the measurement protocols and devices can be found in [46] and is briefly explained in the following sections.

#### 4.3.1. Chlorophyll Fluorescence Imaging

Chlorophyll fluorescence imaging was performed using CropReporter^TM^ (PhenoVation B.V., Wageningen, The Netherlands). The plants were adapted to darkness for 30 min before the measurements, and plants were imaged from a distance of 70 cm. For the excitation of photosynthesis, 4000 µmol m^−2^s^−1^ red LED light was used. The integration time for acquiring the chlorophyll fluorescence image was 200 µs. The minimum chlorophyll fluorescence (F_0_) was measured after ten µs, and the maximum chlorophyll fluorescence (F_m_) was measured after saturation. After measuring the dark-adapted plants, the plants were relaxed in the dark for 15 s, and then actinic light (250 µmol m^−2^ s^−1^) was switched on to allow the plants to adapt to the light for 5 min. The steady-state fluorescence yield (F_s_′) was measured at the beginning of the saturation pulse, and the maximum chlorophyll fluorescence (F_m_′) of the plants adapted to the light was measured at saturation, using the intensity of the saturation pulse (4000 µmol m^−2^s^−1^). After the measurement, the actinic light was switched off, and the minimum fluorescence yield of the illuminated plant (F_0_′) was estimated in the presence of far-red light.

The measured F_0_, F_m_, F_m_′, and F_s_′ were used to calculate the following fluorescence parameters [55,56,57,58,59]:

The maximum quantum yield of PSII (F_v_/F_m_): F_v_/F_m_ = (F_m_ − F_0_)/F_m_.

The effective quantum yield of PSII (F_q_′/F_m_′): F_q_′/F_m_′ = (F_m_′ − F_s_′)/F_m_′.

Electron transport rate (ETR) = F_q_′/F_m_′ × PPFD × (0.5) × 0.83.

Non-photochemical quenching (NPQ) = (F_m_ − F_m_′)/F_m_′.

Coefficient of photochemical quenching (qP) = (F_m_′ − F_s_′)/F_v_.

Coefficient of non-photochemical quenching (qN) = 1 − (F_m_′ − F_0_′)/(F_m_ − F_0_).

Estimation of “open” reaction centers based on a lake model (qL) = ((F_m_′ − F_s_′) × F_0_′))/((F_m_′ − F_0_′) × F_s_′)).

Quantum yield of non-regulated non-photochemical energy loss in PSII (ɸno) = 1/(NPQ + 1 + qL(F_m_/F_0_ − 1))

#### 4.3.2. Multispectral Imaging

After chlorophyll fluorescence imaging, reflectance (R) images were acquired at 250 µmol m^−2^ s^−1^ illumination. Multispectral images were taken at red (R_Red_—640 nm), green (R_Green_—550 nm), blue (R_Blue_—475 nm), specific green (R_SpcGrn_—510–590 nm), chlorophyll reflectance (R_Chl_—730 nm), near-infrared (R_NIR_—769 nm), and far-red reflectance (R_FarRed_—710 nm). The chlorophyll index (CHI), anthocyanin index (ARI), hue (0–360°), saturation (SAT), and value (VAL) were calculated from the measured reflectance [60,61]:

Chlorophyll index (CHI) = (R_Chl_)^−1^ − (R_NIR_)^−1^.

Anthocyanin index ARI = (R_Green_)^−1^ − (R_FarRed_)^−1^.

Hue (0–360◦) was calculated as follows:

HUE = 60 × [0 + (R_Green_ − R_Blue_)/(max − min)], if max = R_Red_;

HUE = 60 × [2 + (R_Blue_ − R_Red_)/(max − min)], if max = R_Green_;

HUE = 60 × [4 + (R_Red_ − R_Green_)/(max − min)], if max = R_Blue_.

360 was added in case of HUE < 0

Value (0–1) was calculated as: 

VAL = (max + min)/2, while max and min were selected from the R_Red_, R_Green_, and R_Blue_, respectively. 

Saturation (0–1) was calculated as follows: 

SAT = (max − min)/(max + min) if VAL > 0.5, or SAT = (max − min)/(2.0 − max − min) if VAL < 0.5, while max and min were selected from the R_Red_, R_Green_, and R_Blue_, respectively.

**Figure 4:**
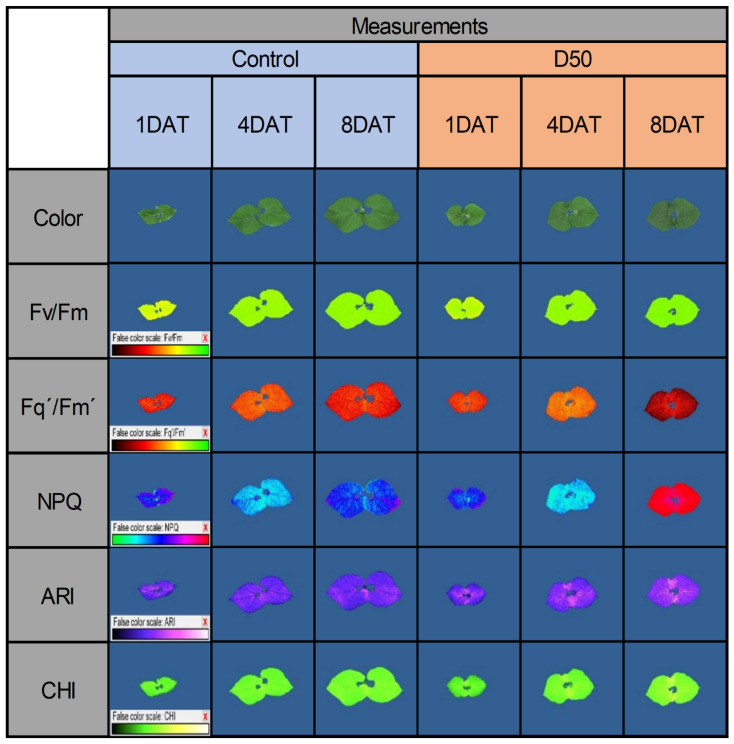
Common bean color and pseudo-color images showing the maximum quantum yield of PSII (Fv/Fm), the effective quantum yield of PSII (Fq′/Fm′), non-photochemical quenching (NPQ), the anthocyanin index (ARI), and the chlorophyll index (CHI) captured by CropReporter on the first (1 DAT), fourth (4 DAT) and eighth (8 DAT) day of the experiment in control and under drought treatment (D50).

Examples of generated chlorophyll fluorescence and multispectral images are shown in Figure 4. 

#### 4.3.3. Multispectral 3D Scanning

The plants were scanned with a multispectral 3D scanner PlantEye F500 (Phenospex, Heerlen, Netherlands) from a distance of 70 cm. PlantEye measures spectral reflectance in red (620–645 nm), green (530–540 nm), blue (peak wavelength 460–485 nm), near-infrared (820–850 nm), and the 3D laser (940 nm). Morphological parameters and vegetation indices were calculated with the software HortControl (Phenospex, Heerlen, The Netherlands).

The vegetation indices calculated are [62,63]:

Green Leaf Index (GLI) = (2 × R_Green_ − R_Red_ − R_Blue_)/(2 × R_Green_ + R_Red_ + R_Blue_).

Normalized Differential Vegetation Index (NDVI) = (R_NIR_ − R_Red_)/(R_NIR_ + R_Red_)

Plant Senescence Reflectance Index (PSRI) = (R_Red_ − R_Green_)/(R_NIR_).

The morphological parameters calculated from the 3D plant model were: Plant height (P.H.; mm)—calculated as the distribution of elementary triangles along the *z*-axis; leaf area projected (LAP; cm^2^)—calculated as the area of the projection of all elementary triangles onto the X-Y plane; total leaf area (TLA; cm^2^)—calculated as the sum of all triangle domains, where each domain represents a group of triangles forming a unitary area; digital volume (DV; cm^3^)—calculated as the product of the height and 3D leaf area; leaf area index (LAI, mm^2^ mm^−2^)—calculated as TLA/sector size; leaf inclination (LINC; mm^2^ mm^−2^)—describing how erect the leaves of the plant are and calculated as TLA/LAP; leaf angle [LANG; degree (°)]; and light penetration depth (LPD; mm)—measured by the deepest point at which the laser can penetrate the canopy along the *z*-axis.

### 4.4. Statistical Analyses

The normality of the collected data was tested by checking the residual plots and homogeneity of variance (homoscedasticity) by plotting residuals against fitted values. The analysis of variance (ANOVA) with repeated measures was performed as described by [64], using the MIXED procedure in JMP^®^ Pro 16 (SAS Institute Inc., Cary, NC, USA). The model included the fixed effects of treatment (control and drought treatments D30, D50, and D70), measurement time (1 DAT–5 DAT, and 8 DAT used for repeated measures), and treatment × measurement time interaction. Individual plants (subjects) were treated as a random factor and were nested within the treatments. The autoregressive (AR) covariance structure model was chosen among AR, compound symmetry, and unstructured model, based on the Akaike information criterion (AICc) that smaller is better. Tukey’s Honest Significant Difference post hoc test was performed for partitioned F-tests (SLICE option) to examine the significance of treatment differences within measurement times. 

## 5. Conclusions

As drought becomes more frequent in severe forms, it is recognized as one of the major threats to global food security. The improvement of crop germplasm through breeding can address this problem, but it needs to be supported by reliable phenotyping data. In this study, using non-destructive phenotyping, we have quantified and extracted phenotypic traits important for the early detection of drought stress in the common bean. Most sensitive phenotypic traits under drought stress were related to leaf area changes and spectral characteristics. Namely, the earliest detected changes under drought stress were a decrease in the LAI and TLA, an increase in the anthocyanin index (ARI) and R_Blue_, and a decrease in the GLI, SAT, and R_SpcGrn_. These early changes probably protected leaf photochemistry, as chlorophyll fluorescence traits were found to be affected by drought only after prolonged drought stress. The selected phenotypic traits could be used to monitor drought stress and could be used for screening for drought-tolerant genotypes in breeding programs.

## Data Availability

The data that support the findings of this study are available from the corresponding author [T.J.] upon reasonable request.

## Supplementary Materials

The following supporting information can be downloaded at: https://www.mdpi.com/article/10.3390/plants12061386/s1, Table S1: The results of the repeated measures of ANOVA and ANOVA test with SLICE option. Table S2: The least-square means for partitioned F-tests (SLICE option) to examine the significance of treatments within time. Table S3: Average readings of the sand volumetric water content (VWC) at each measurement time. Table S4: The list of all measured plant traits, the abbreviation, and the devices used for the measurement.
